# 346. Aspergillosis in COVID-19 era in Dominican Republic

**DOI:** 10.1093/ofid/ofac492.424

**Published:** 2022-12-15

**Authors:** Rita A Rojas-Fermin, Ann S Sanchez-Marmolejos, Ricardo Acra-Tolari, Anel E Guzman-Marte, Irina Suero

**Affiliations:** Hospital General de la Plaza de la Salud, Santo Domingo, Distrito Nacional, Dominican Republic; Hospital General Plaza de la Salud, Santo Domingo, Distrito Nacional, Dominican Republic; Hospital General de la Plaza de la Salud, Santo Domingo, Distrito Nacional, Dominican Republic; Hospital General de la Plaza de la Salud, Santo Domingo, Distrito Nacional, Dominican Republic; Hospital General de la Plaza de la Salud, Santo Domingo, Distrito Nacional, Dominican Republic

## Abstract

**Background:**

COVID-19-associated Pulmonary Aspergillosis (CAPA) and Invasive Pulmonary Aspergillosis (IPA) represent a difficult diagnostic challenge to the clinician. Moreover, during the COVID-19 pandemic, as airway invasive procedures were limited due to fear of contamination, these diagnoses were even harder to make, as one of the most useful diagnosis tools, bronchoscopies, were postponed. The aim of this study was to describe the epidemiology and risks factors of Aspergillosis and CAPA in a Dominican tertiary health care facility during the COVID-19 pandemic.

**Table 1:**
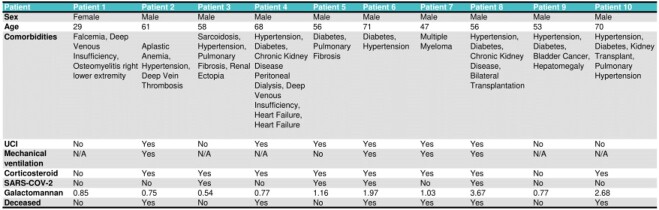

Characteristics of patients with COVID-19-associated Pulmonary Aspergillosis (CAPA) and Invasive Pulmonary Aspergillosis (IPA)

**Methods:**

A retrospective, cross-sectional, case-series study was carried out were all patients during the COVID-19 pandemic from March 2020 - March 2022 in HGPS who had a galactomannan (GM) test were analyzed. Using E-records patients with positive galactomannan (GM) tests were identified, and the following variables were evaluated: demographics, laboratories, risk factors, comorbidities, CT scans and prognosis. Cases were classified as CAPA (Probable, possible or proven according to classification ECMM/ISHAM), or IPA.

**Results:**

Out of 77 patients who underwent a GM test, 10 had a positive result; 40% of these were probable CAPA and 60% were IPA. 7 were co-infected with multidrug-resistant pathogens from which 71.4% died; overall median age was 56.9 years (minimum 29 – maximum 71), and only one patient was female. The overall mortality rate was 60% and 50% for the probable CAPA group. Most radiological findings from the probable CAPA group were classified as typical invasive pulmonary aspergillosis. Major documented risk factors and comorbidities were lymphopenia, prolonged steroids use, hypertension, diabetes mellitus and mechanical ventilation.

**Conclusion:**

An increase in both CAPA and IPA screening is needed in patients who present risk factors such as mechanical ventilation, prolonged use of steroids, or renal replacement therapy. Screening for GM in bronchoalveolar lavage, mycologic cultures and histopathologic tests, are needed for an improvement in the diagnostic classification as well as the clinical outcome of these patients.

**Disclosures:**

**Rita A. Rojas-Fermin, MD**, Gilead: Advisor/Consultant|MSD: Expert Testimony|MSD: Honoraria|Pfizer: Advisor/Consultant.

